# Multiplex Real-Time PCR Assay Using TaqMan Probes for the Identification of *Trypanosoma cruzi* DTUs in Biological and Clinical Samples

**DOI:** 10.1371/journal.pntd.0003765

**Published:** 2015-05-19

**Authors:** Carolina I. Cura, Tomas Duffy, Raúl H. Lucero, Margarita Bisio, Julie Péneau, Matilde Jimenez-Coello, Eva Calabuig, María J. Gimenez, Edward Valencia Ayala, Sonia A. Kjos, José Santalla, Susan M. Mahaney, Nelly M. Cayo, Claudia Nagel, Laura Barcán, Edith S. Málaga Machaca, Karla Y. Acosta Viana, Laurent Brutus, Susana B. Ocampo, Christine Aznar, Cesar A. Cuba Cuba, Ricardo E. Gürtler, Janine M. Ramsey, Isabela Ribeiro, John L. VandeBerg, Zaida E. Yadon, Antonio Osuna, Alejandro G. Schijman

**Affiliations:** 1 Laboratorio de Biología Molecular de la Enfermedad de Chagas, Instituto de Investigaciones en Ingeniería Genética y Biología Molecular “Dr. Héctor N. Torres”—INGEBI-CONICET, Buenos Aires, Argentina; 2 Instituto de Medicina Regional, Universidad Nacional del Nordeste, Resistencia, Chaco, Argentina; 3 Laboratoire Hospitalier et Universitaire-CH Andrée Rosemon, Cayenne, French Guiana, France; 4 Laboratorio Biología Celular, Centro de Investigaciones Regionales “Dr. Hideyo Noguchi”, Universidad Autónoma de Yucatán, Mérida, Yucatán, Mexico; 5 Servicio de Medicina Interna, Hospital Politécnico LA FE, Valencia, Spain; 6 Servicio de Microbiología, Hospital Universitario y Politécnico LA FE, Valencia, Spain; 7 Laboratorio de Investigación en Enfermedades Infecciosas, Universidad Peruana Cayetano Heredia, Lima, Peru; 8 Department of Biology, University of Minnesota Duluth, Duluth, Minnesota, United States of America; 9 Laboratorio de Parasitología, Instituto Nacional de Laboratorios en Salud, Ministerio de Salud y Deportes de Bolivia, La Paz, Bolivia; 10 Southwest National Primate Research Center and Department of Genetics, Texas Biomedical Research Institute, San Antonio, Texas, United States of America; 11 Instituto de Biología de la Altura, Universidad Nacional de Jujuy, Jujuy, Argentina; 12 Epidemiología e Infectología Clínica, Hospital Universitario Fundación Favaloro, Buenos Aires, Argentina; 13 Sección Infectología, Servicio de Clínica Médica, Hospital Italiano, Buenos Aires, Argentina; 14 Institut de Recherche pour le Développement and University Paris Descartes, UMR 216, Mother and Child Facing Tropical Diseases, Paris, France; 15 Parasitologia Médica e Biologia de Vetores, Área de Patologia, Faculdade de Medicina, Universidade de Brasilia, Brasilia DF, Brazil; 16 Laboratorio de Eco-Epidemiología, Departamento de Ecología, Genética y Evolución, Facultad de Ciencias Exactas y Naturales, Universidad de Buenos Aires, Buenos Aires, Argentina; 17 Centro Regional de Investigación en Salud Pública, Instituto Nacional de Salud Pública, Tapachula, Chiapas, Mexico; 18 Drugs and Neglected Diseases Initiative, Genève, Switzerland; 19 Pan American Health Organization (PAHO), World Health Organization (WHO), Washington, D.C., United States of America; 20 Institute of Biotechnology, Molecular Parasitology Group, University of Granada, Granada, Spain; US Food and Drug Administration, UNITED STATES

## Abstract

**Background:**

*Trypanosoma cruzi* has been classified into six Discrete Typing Units (DTUs), designated as TcI–TcVI. In order to effectively use this standardized nomenclature, a reproducible genotyping strategy is imperative. Several typing schemes have been developed with variable levels of complexity, selectivity and analytical sensitivity. Most of them can be only applied to cultured stocks. In this context, we aimed to develop a multiplex Real-Time PCR method to identify the six *T*. *cruzi* DTUs using TaqMan probes (MTq-PCR).

**Methods/Principal Findings:**

The MTq-PCR has been evaluated in 39 cultured stocks and 307 biological samples from vectors, reservoirs and patients from different geographical regions and transmission cycles in comparison with a multi-locus conventional PCR algorithm. The MTq-PCR was inclusive for laboratory stocks and natural isolates and sensitive for direct typing of different biological samples from vectors, reservoirs and patients with acute, congenital infection or Chagas reactivation. The first round SL-IR MTq-PCR detected 1 fg DNA/reaction tube of TcI, TcII and TcIII and 1 pg DNA/reaction tube of TcIV, TcV and TcVI reference strains. The MTq-PCR was able to characterize DTUs in 83% of triatomine and 96% of reservoir samples that had been typed by conventional PCR methods. Regarding clinical samples, 100% of those derived from acute infected patients, 62.5% from congenitally infected children and 50% from patients with clinical reactivation could be genotyped. Sensitivity for direct typing of blood samples from chronic Chagas disease patients (32.8% from asymptomatic and 22.2% from symptomatic patients) and mixed infections was lower than that of the conventional PCR algorithm.

**Conclusions/Significance:**

Typing is resolved after a single or a second round of Real-Time PCR, depending on the DTU. This format reduces carryover contamination and is amenable to quantification, automation and kit production.

## Author Summary

Chagas disease, caused by the protozoan *Trypanosoma cruzi*, represents a health and social threat to an estimated number of eight million people, affecting mainly neglected populations in endemic areas and emerging in non endemic countries by migratory movements. Parasite genetic diversity is related to geographical distribution and transmission cycles and might play a role in clinical manifestations as well as in anti-parasitic chemotherapy response. *T*. *cruzi* has been classified into six Discrete Typing Units (DTUs), after consensus reached among experts in the field. In order to effectively use this standardized nomenclature, a reproducible genotyping strategy is needed. Available typing schemes are usually applied to cultured parasite stocks, because they are not sensitive enough to be used in biological specimens. Only nested PCR procedures could directly type biological samples, but are prompt to contamination and require a high number of reactions. Thus, we developed a multiplex Real-Time PCR using TaqMan probes (MTq-PCR) for DTU typing in a single or a second round of amplification. It proved useful to determine DTUs in cultured stocks, vector and reservoir specimens, as well as in patients´samples, especially in those from individuals with acute, congenital infection or Chagas reactivation. It is amenable to quantification and automation for kit production.

## Introduction

Infection with *Trypanosoma cruzi* is a complex zoonosis, transmitted by more than 130 triatomine species and sustained by over 70 genera of mammalian reservoir hosts. *T*. *cruzi* has a broad endemic range that extends from the Southern United States to Argentinean Patagonia. The human infection, which may lead to Chagas disease, is the most important parasitic infection in Latin America with serious consequences for public health and national economies.

The diversity of the *T*. *cruzi* genome is well recognized [1–3]. Designation of ecologically and epidemiologically relevant groups for *T*. *cruzi* has oscillated between a few discrete groups [4] and many [5]. Currently, six Discrete Typing Units (DTUs) are defined [2]. In 2009, these DTUs were renamed by consensus as TcI–TcVI [6]. Several reviews already describe how these DTUs correspond with former nomenclatures and with prospective biological and host associations [6–8]. All six DTUs are known to be infective to humans and to cause Chagas disease. Further, in patients infected with DTU mixtures, different tissue distribution has been detected [9–11]. Recently a new genotype associated with anthropogenic bats (TcBat) has been detected in Brazil, Panama and Colombia and awaits further characterization for definitive DTU assignment [12–14].

The standardized nomenclature for *T*. *cruzi* DTUs should improve scientific communication and guide future research on comparative epidemiology and pathology. However, a straightforward and reproducible DTU genotyping strategy is still required. Numerous approaches have been proposed to characterize the biochemical and genetic diversity of *T*. *cruzi* isolates [15–23] with variable levels of complexity, selectivity and analytical sensitivity. Due to sensitivity constraints, most of these strategies have been applied only to cultured stocks and not directly to biological or clinical samples. Thus, their results may have underestimated parasite diversity due to possible strain selection during culture expansion [24–25]. Some methods require multiple sequential conventional PCR reactions, PCR-RFLP, hybridization or post-PCR sequencing steps; these tests are cumbersome and time-consuming, and their results are often difficult to interpret. Accordingly, we aimed to develop a novel multiplex Real-Time PCR method using TaqMan probes, allowing distinction of the six DTUs in a few steps not only from cultured stocks but also from a high proportion of biological and clinical samples.

## Materials and Methods

### Biological Samples

Reference strains: Genomic DNA from a panel of reference stocks representative of the 6 *T*. *cruzi* DTUs, *Trypanosoma rangeli* and *Leishmania* spp. was used for analytical validation of the assay (Table 1).

**Table 1:** *T*. *cruzi*, *T*. *rangeli* and *Leishmania* spp. isolates used to evaluate the analytical performance of the multiplex real-time PCR genotyping assays.

Strain	DTU/Species	Origin	Vector/Host
K-98^a^	TcI	Argentina	*Homo sapiens*
PalDa30^b^	TcI	Argentina	*Didelphis albiventris*
SE9V^c^	TcI	Argentina	*Homo sapiens*
TCC^d^	TcI	Chile	*Homo sapiens*
13379 cl7^e^	TcI	Bolivia	*Homo sapiens*
G^a^	TcI	Brazil	*Didelphis marsupialis*
Sylvio X10^a^	TcI	Brazil	*Homo sapiens*
Triatoma^f^	TcI	Mexico	*Triatoma sp*.
Duran^f^	TcI	Mexico	nd
Gamma^f^	TcI	Mexico	nd
Colombiana^a^	TcI	Colombia	*Homo sapiens*
Dm28c^a^	TcI	Venezuela	*Didelphis marsupialis*
OPS21cl11^g^	TcI	Venezuela	*Homo sapiens*
Tu18^a^	TcII	Bolivia	*Triatoma infestans*
Basileu^h^	TcII	Brazil	*Homo sapiens*
Y^a^	TcII	Brazil	*Homo sapiens*
MAS cl1^a^	TcII	Brazil	*Homo sapiens*
Ll51-P24-Ro^i^	TcIII	Argentina	*Canis familiaris*
M5631 cl5^a^	TcIII	Brazil	*Dasypus novemcinctus*
M6241 cl6^a^	TcIII	Brazil	*Homo sapiens*
3663^a^	TcIII	Brazil	*Panstrongylus geniculatus*
X109/2^a^	TcIII	Paraguay	*Canis familiaris*
CanIII^a^	TcIV	Brazil	*Homo sapiens*
4167^a^	TcIV	Brazil	*Rhodnius brethesi*
Griffin^j^	TcIV	USA	*Canis familiaris*
Dog Theis^a^	TcIV	USA	*Canis familiaris*
92122102R^a^	TcIV	USA	*Procyon lotor*
PAH265^k^	TcV	Argentina	*Homo sapiens*
PAH179^k^	TcV	Argentina	*Homo sapiens*
LL014-1R1cl1^l^	TcV	Argentina	nd
MN cl2^a^	TcV	Chile	*Homo sapiens*
SO3 cl5^a^	TcV	Bolivia	*Triatoma infestans*
RA^a^	TcVI	Argentina	*Homo sapiens*
Tep7^k^	TcVI	Argentina	*Canis familiaris*
Tep6 cl5^k^	TcVI	Argentina	*Canis familiaris*
LL052^m^	TcVI	Argentina	nd
Tulahuen cl2^a^	TcVI	Chile	*Homo sapiens*
CL Brener^a^	TcVI	Brazil	*Triatoma infestans*
Peruana^n^	TcVI	Perú	nd
444^o^	*T*. *rangeli*	Colombia	*Rhodnius prolixus*
SC-58^p^	*T*. *rangeli*	Brazil	*Echimys dasythrix*
Tre^q^	*T*. *rangeli*	Colombia	nd
L1566^r^	*L*. *major*	Ecuador	*Homo sapiens*
M2269^s^	*L*. *amazonensis*	Brazil	*Homo sapiens*
L1569^r^	*L*. *brasiliensis*	Ecuador	*Homo sapiens*
L1508^r^	*L*. *mexicana*	Belize	*Homo sapiens*

References: ^a^[6]; ^b^[26]; ^c^[27]; ^d^[28]; ^e^[29]; ^f^[30]; ^g^[31]; ^h^[32]; ^i^[33] ^j^[34]; ^k^[22]; ^l^[23]; ^m^[35]; ^n^[36]; ^o^[37]; ^p^[38]; ^q^[39]; ^r^[40]; ^s^[41]. DTU, Discrete Typing Unit; nd, no data.

Clinical specimens: A total of 132 clinical samples were included in the study: one tissue sample and 131 peripheral blood samples obtained from acute *T*. *cruzi* infected patients (AI, n = 13), asymptomatic (ACD, n = 64) and symptomatic (SCD, n = 27, 19 cardiac, 5 digestive and 3 mixed disease patients) chronic Chagas disease patients, congenitally infected children (CI, n = 16), and from adult patients with clinical reactivation in the context of immunosuppression (RCD, n = 11) (S1 Table).

Triatomine samples: A total of 104 triatomine derived samples were included in the study: 16 culture isolates and 88 direct samples (38 abdomen/midgut samples and 50 feces/urine samples collected on filter paper) from infected bugs (S2 Table).

Mammalian reservoir samples: A total of 71 samples obtained from *T*. *cruzi* reservoirs were included in the study: 27 culture isolates and 44 direct samples (38 peripheral blood samples and 6 heart explants) from mammalian reservoirs (S3 Table).

### Ethics Statement

The study with human samples was approved by the ethical committees of the participating institutions (Comité de Bioseguridad del INLASA, Ministerio de Salud de Bolivia; Comité de Ética en Investigación de la Universidad de Granada; Comité de Ética de Investigación del Instituto Nacional de Salud Pública de México; Comité de Ética del Hospital Italiano; Comité de Bioética del Hospital Universitario Fundación Favaloro; Comité de Bioética del Instituto de Medicina Regional de la Universidad Nacional del Nordeste; Comité de Bioética de la provincia de Jujuy), following the principles expressed in the Declaration of Helsinki. Written informed consents were obtained from the adult patients and from parents/guardians on behalf of all children participants.

### DNA Extraction

Preparation of DNA from biological specimens was done according to the type of sample and the operating procedures followed by the laboratories from which DNA aliquots were obtained (S1–S3 Tables). At our laboratory, peripheral blood and tissue samples were processed using High Pure PCR Template Preparation Kit (Roche, Germany) following the recommendations of the manufacturer. Triatomine feces impregnated on filter paper and abdomen samples were processed as reported [42].

### Conventional PCR Based Discrete Typing Unit Genotyping

Identification of *T*. *cruzi* DTUs was assessed using a conventional PCR algorithm for DTU genotyping, based on the amplification of three nuclear loci, the spliced leader intergenic region (SL-IR), the 24Sα-ribosomal DNA (24Sα-rDNA) and the A10 fragment, as reported [11,17]. Analytical sensitivity for these methods was described in Burgos et al. (2007) [17]: SL-IRac PCR: 1 pg, SL-IR I PCR: 5 pg, SL-IR II PCR: 5 pg, 24Sα-rDNA PCR: 100 fg, and A10 PCR: 1–10 pg DNA per reaction tube.

### TaqMan Probes and Primer Design

Multiple sequence alignments of the *T*. *cruzi* SL-IR, cytochrome oxidase subunit II (COII), 18S ribosomal DNA (18S rDNA) and 24Sα-rDNA genes were performed using the ClustalW algorithm in MEGA 5.2 software [43]. Reference sequences were retrieved from the GenBank database. The PrimerQuest and OligoAnalyzer tools (provided online at the website http://www.idtdna.com) were used for the final design of specific primers and probes (Table 2). To minimize nonspecific detection, the oligonucleotides were compared with all relevant sequences using the BLAST database search program (provided online from the National Center for Biotechnology Information [NCBI]).

**Table 2:** Sequences and concentrations of primers and probes used in the multiplex real-time PCR assays.

PCR assay	Oligonucleotide	Sequence (5'- 3')	Final concentration (μM)
**SL-IR MTq**	UTCC-Fw	CAGTTTCTGTACTATATTGGTACG	0.5
	TcI-Rv	CGATCAGCGCCACAGAAAGT	0.5
	TcII/V/VI-Rv	GGAAAACACAGGAAGAAGC	0.5
	TcIII-Rv	CATTTTTATGAGGGGTTGTTCG	0.5
	TcIV-Rv	CATTTTTATTAGGGGTTGTACG	0.5
	TcI (probe)	FAM-CTC+CTTC+AT+GTT+TGT+GTCG-BHQ1	0.1
	TcII/V/VI (probe)	HEX-TATA+CC+CATATA+TATA+TA+GC-BHQ1	0.05
	TcIII (probe)	Quasar670-AATCGCG+TGTATGCACCGT-BHQ3	0.05
	TcIV (probe)	CAL Fluor Red610-GCCCCCGACGCCGTCCGTG-BHQ2	0.1
**18S-COII MTq**	18S-Fw	ATGGGATAACAAAGGAGCAGCCTC	0.2
	18S-Rv	CTTCATTCCTGGATGCCGTGAGTT	0.2
	COII-Fw	ACACCTACCYGGTTCTCTACCT	0.2
	COII-Rv	CTYGARAGTGATTAYTTGGTGGGWG	0.2
	18S-TcII/VI (probe)	FAM-CAGACTTCGGTCTTACCCTTCGCATCTCACA-BHQ1	0.05
	18S-TcV (probe)	HEX-TCTT+GCC+T+C+CGCATATTTTCACA-BHQ1	0.05
	COII-TcII (probe)	Cy5-AATGGATTACATCTACGGCTGACACCCA-BHQ3	0.1
**24Sα-III/IV MTq**	D71^a^	AAGGTGCGTCGACAGTGTGG	0.4
	D76^b^	GGTTCTCTGTTGCCCCTTTT	0.4
	TcIII (probe)	FAM-CTTTTCC+C+C+TCTCTTTTATTA+GG-BHQ1	0.2
	TcIV (probe)	HEX-+T+G+CTCTCTTTCCTTCTCTT+TACG-BHQ1	0.2

^a^[44]; ^b^[45]; SL-IR, spliced leader intergenic region; 18S, 18S-ribosomal ADN; COII, cytochrome oxidase II; 24Sα, 24Sα-ribosomal ADN; MTq, multiplex Real-Time PCR; BHQ, Black Hole Quencher. The + in front of the nucleotide indicates an LNA (Locked Nucleic Acid) monomer substitution.

### Multiplex Real-Time PCR Assays

**Fig 1:**
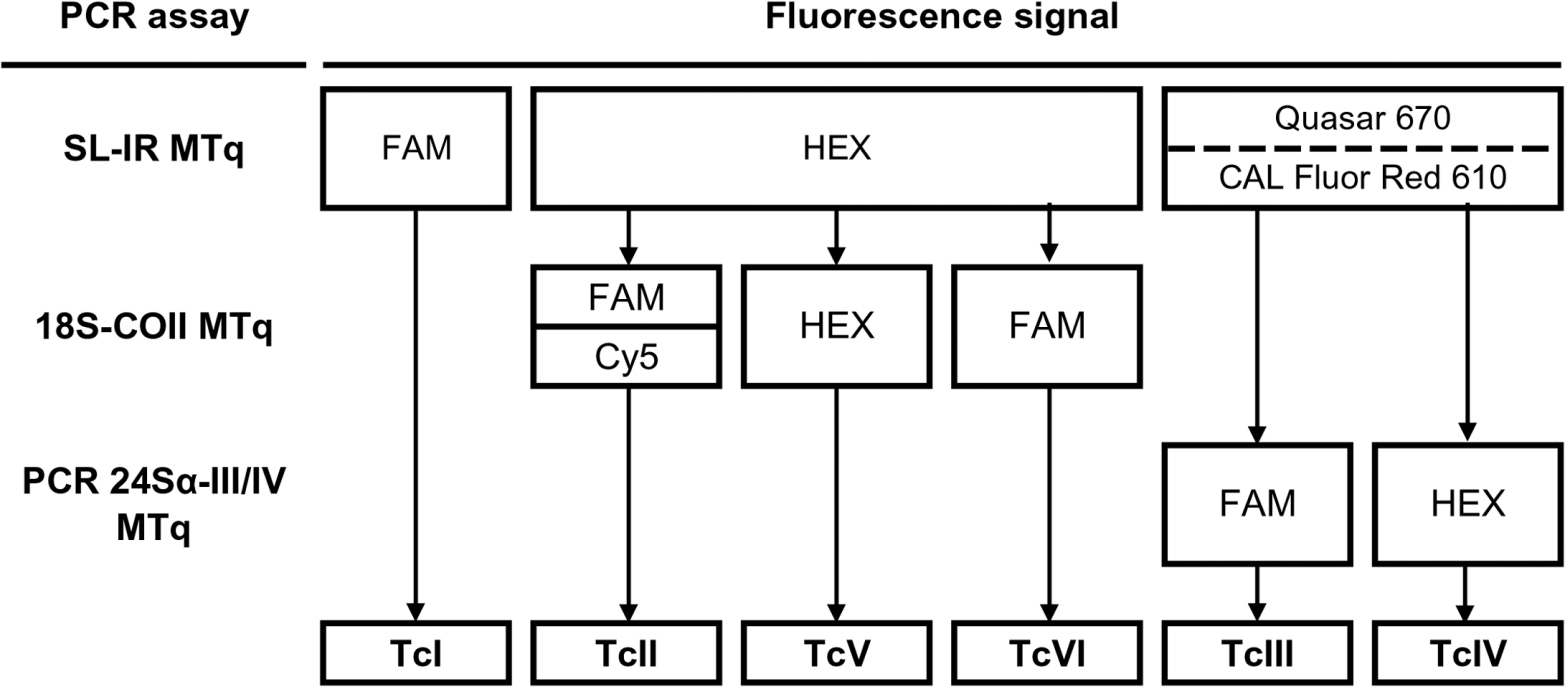
Multiplex real-time PCR flowchart for identification of *Trypanosoma cruzi* DTUs in biological samples. SL-IR, spliced leader intergenic region; 18S, 18S-ribosomal ADN; COII, cytochrome oxidase II; 24Sα, 24Sα-ribosomal DNA; MTq, multiplex TaqMan Real-Time PCR.

A Real-Time PCR flowchart for identification of *T*. *cruzi* DTUs in biological samples using TaqMan probes (MTq-PCR) is shown in Fig 1. Oligonucleotide concentration and sequence information is detailed in Table 2. TaqMan probes were purchased from Integrated DNA Technologies, Inc. (USA). SL-IR and 18S-COII MTq-PCR assays were carried out using 1X QIAGEN Multiplex PCR Kit (QIAGEN, USA), while the 24Sα-III/IV MTq-PCR used 1X FastStart Universal Probe Master (Roche, Germany). All PCR reactions were carried out with 2 μL of resuspended DNA in a final volume of 20 μL. Optimal cycling conditions for the SL-IR and 18S-COII MTq-PCR assays were initially 15 min at 95°C followed by 40 cycles at 95°C for 30 sec and 60°C for 1 min in an Applied Biosystems (ABI 7500, USA) device. In turn, optimal cycling condition for the 24Sα-III/IV reaction was an initial cycle of 10 min at 95°C followed by 40 cycles at 95°C for 30 sec and 57°C for 1 min in a Rotor-Gene 6000 (Corbett, UK) device.

### Analytical Performance of the Multiplex Real-Time PCR Assays

In order to characterize the performance of the MTq-PCR, several analytical parameters were determined [40].

The inclusivity of the assays was evaluated using 0.05–5 ng/μL of genomic DNA obtained from a panel of 39 *T*. *cruzi* stocks belonging to the six DTUs from different geographic origins (Table 1). On the other hand, 1–5 ng/μL of genomic DNA obtained from *T*. *rangeli*, *L*. *major*, *L*. *amazonensis*, *L*. *brasiliensis* and *L*. *mexicana*, was used to assess the specificity of the assays. Specificity was also tested using human DNA from a seronegative patient as template.

Analytical sensitivity and reaction efficiency were evaluated using 2-fold, 10-fold and 100-fold serial dilutions spanning 1 μg to 1 fg of genomic DNA per reaction tube obtained from *T*. *cruzi* stocks belonging to different DTUs, depending on the assay. Moreover, in the case of TcI, four stocks representing TcIa, TcIb, TcId and TcIe genotypes based on the polymorphism of the SL-IR gene were analyzed [30]. In addition, in the case of TcIV, DNA from strains representing populations from South America (TcIV-SA) and North America (TcIV-NA) were used [46]. Each concentration was tested in duplicate.

## Results

### Analytical Performance of the Multiplex Real-Time PCR Assays

Fig 1 illustrates the MTq-PCR flowchart designed to distinguish among the six *T*. *cruzi* DTUs.

Inclusivity and specificity results are shown in Table 3. *T*. *cruzi* I, including stocks representing SL-IR genotypes TcIa, TcIb, TcId and TcIe, were detected by the FAM fluorescence signal in the SL-IR MTq-PCR assay and did not amplify in the downstream reactions of the flowchart. The TcII/V/VI group was detected with the HEX-labeled probe in the SL-IR MTq-PCR. The 18S-COII MTq-PCR assay distinguished TcII (FAM + Cy5 signals) from TcV (HEX signal) and TcVI (FAM signal only). There were two groups of TcIII strains, one group reacted only with the SL-IR TcIII-Quasar670 probe, and the other one composed by three strains (from Brazil, Paraguay and Argentina), reacted with both TcIII-Quasar670 and TcIV-CAL Fluor Red610 SL-IR probes. Thus, the latter group of strains was identified as TcIII after a second round of amplification using the 24Sα-FAM probe. CAL Fluor Red610 and HEX fluorescence signals were detected when the assay contained DNA from TcIV-SA and TcIV-NA strains in the SL-IR and the 24Sα-III/IV MTq-PCR assays, respectively.

**Table 3:** Inclusivity and specificity assays for the multiplex real-time PCR genotyping algorithm.

Strain	Species	DTU	SL-IR MTq PCR assay				18S-COII MTq PCR assay			24Sα-III/IV MTq PCR assay	
			TaqMan probe				TaqMan probe			TaqMan probe	
			TcI FAM	TcII/V/VI HEX	TcIII Quas670	TcIV Cal610	18S-TcII/VI FAM	18S-TcV HEX	COII-TcII Cy5	TcIII FAM	TcIV HEX
G	*T*. *cruzi*	TcI	**14.11**	neg	neg	neg	neg	neg	neg	neg	neg
K-98	*T*. *cruzi*	TcI	**17.98**	neg	neg	nd	nd	nd	nd	nd	nd
PalDa30	*T*. *cruzi*	TcI	**22.97**	neg	neg	nd	nd	nd	nd	nd	nd
SE9V	*T*. *cruzi*	TcI	**21.04**	neg	neg	nd	nd	nd	nd	nd	nd
TCC	*T*. *cruzi*	TcI	**22.52**	neg	neg	nd	nd	nd	nd	nd	nd
13379 cl7	*T*. *cruzi*	TcI	**38.68**	neg	neg	nd	nd	nd	nd	nd	nd
Sylvio X10	*T*. *cruzi*	TcI	**12.53**	neg	neg	nd	nd	nd	nd	nd	nd
Triatoma	*T*. *cruzi*	TcI	**13.93**	neg	neg	nd	nd	nd	nd	nd	nd
Duran	*T*. *cruzi*	TcI	**11.10**	neg	neg	nd	nd	nd	nd	nd	nd
Gamma	*T*. *cruzi*	TcI	**11.42**	neg	neg	nd	nd	nd	nd	nd	nd
Colombiana	*T*. *cruzi*	TcI	**23.35**	neg	neg	nd	nd	nd	nd	nd	nd
Dm28c	*T*. *cruzi*	TcI	**16.69**	neg	neg	nd	nd	nd	nd	nd	nd
OPS21cl11	*T*. *cruzi*	TcI	**35.52**	neg	neg	nd	nd	nd	nd	nd	nd
Tu18	*T*. *cruzi*	TcII	neg	**15.16**	neg	neg	**19.79**	neg	**19.24**	neg	neg
Basileu	*T*. *cruzi*	TcII	neg	**29.05**	neg	neg	**23.38**	neg	**26.15**	nd	nd
Y	*T*. *cruzi*	TcII	neg	**26.14**	neg	neg	**20.92**	neg	**20,71**	nd	nd
MAS cl1	*T*. *cruzi*	TcII	neg	**16.31**	neg	neg	**20.15**	neg	**19,13**	nd	nd
M5631	*T*. *cruzi*	TcIII	neg	neg	**18.55**	neg	neg	**17.07**	neg	**20.32**	neg
Ll51-P24-Ro	*T*. *cruzi*	TcIII	neg	neg	**33.67**	**32.59**	nd	nd	nd	**25.96**	neg
M6241 cl6	*T*. *cruzi*	TcIII	neg	neg	**20.17**	neg	nd	nd	nd	**18.34**	neg
3663	*T*. *cruzi*	TcIII	neg	neg	**38.75**	**34.21**	nd	nd	nd	**25.14**	neg
X109/2	*T*. *cruzi*	TcIII	neg	neg	**31.26**	**23.34**	nd	nd	nd	**17.96**	neg
CanIII	*T*. *cruzi*	TcIV	neg	neg	neg	**17.62**	neg	**38.19**	neg	neg	**24.42**
4167	*T*. *cruzi*	TcIV	neg	neg	neg	**15.44**	nd	nd	nd	neg	**23.15**
Griffin	*T*. *cruzi*	TcIV	neg	neg	neg	**33.57**	nd	nd	nd	neg	**31.98**
Dog Theis	*T*. *cruzi*	TcIV	neg	neg	neg	**14.12**	nd	nd	nd	neg	**23.84**
92122102R	*T*. *cruzi*	TcIV	neg	neg	neg	**13.92**	nd	nd	nd	neg	**25.57**
PAH265	*T*. *cruzi*	TcV	neg	**24.09**	neg	neg	neg	**24.11**	neg	**27.39**	neg
PAH179	*T*. *cruzi*	TcV	neg	**20.46**	neg	neg	neg	**20.39**	neg	nd	nd
LL014-1R1cl1	*T*. *cruzi*	TcV	neg	**33.15**	neg	neg	neg	**34.64**	neg	nd	nd
MN cl2	*T*. *cruzi*	TcV	neg	**20.83**	neg	neg	neg	**18.67**	neg	nd	nd
SO3 cl5	*T*. *cruzi*	TcV	neg	**26.06**	neg	neg	neg	**26.32**	neg	nd	nd
CL Brener	*T*. *cruzi*	TcVI	neg	**16.49**	neg	neg	**21.92**	neg	neg	neg	neg
RA	*T*. *cruzi*	TcVI	neg	**27.88**	neg	neg	**27.56**	neg	neg	nd	nd
Tep7	*T*. *cruzi*	TcVI	neg	**20.20**	neg	neg	**19.24**	neg	neg	nd	nd
Tep6 cl5	*T*. *cruzi*	TcVI	neg	**20.31**	neg	neg	**21.63**	neg	neg	nd	nd
LL052	*T*. *cruzi*	TcVI	neg	**28.01**	neg	neg	**30.82**	neg	neg	nd	nd
Tulahuen cl2	*T*. *cruzi*	TcVI	neg	**35.15**	neg	neg	**36.16**	neg	neg	nd	nd
Peruana	*T*. *cruzi*	TcVI	neg	**29.6**	neg	neg	**29.23**	neg	neg	nd	nd
444	*T*. *rangeli*	-	neg	neg	neg	neg	neg	neg	neg	neg	neg
SC-58	*T*. *rangeli*	-	neg	neg	neg	neg	neg	neg	neg	neg	neg
Tre	*T*. *rangeli*	-	neg	neg	neg	neg	neg	neg	neg	neg	neg
Lmex	*Leishmania mexicana*	-	neg	neg	neg	neg	neg	neg	neg	neg	neg
La	*Leishmania amazonensis*	-	neg	neg	neg	neg	neg	neg	neg	neg	neg
Lm	*Leishmania major*	-	neg	neg	neg	neg	neg	neg	neg	neg	neg
Lb	*Leishmania brasiliensis*	-	neg	neg	neg	neg	neg	neg	neg	neg	neg
Human DNA	*Homo sapiens*	-	neg	neg	neg	neg	neg	neg	neg	neg	neg

Cycle threshold (Ct) values obtained for each TaqMan probe in the analysis of *T*. *cruzi*, *T*. *rangeli* and *Leishmania* sp. stocks and human DNA. 0.1–10 ng of each *T*. *cruzi* strain and 2–10 ng of *T*. *rangeli* and *Leishmania* spp. stocks were used in the reaction tube.

DTU, Discrete Typing Unit; neg, negative; nd, not done.

TcV was amplified and detected with the FAM probe in the 24Sα-III/IV MTq-PCR assay. Besides, TcIII and TcIV were also detected with the 18S-HEX probe in the 18S-COII MTq-PCR. Specificity of the MTq-PCR was not affected since all these DTUs are confirmed in a previous stage.

On the other hand, MTq-PCR was tested with purified DNA from *T*. *rangeli*, *L*. *amazonensis*, *L*. *major* and *L*. *mexicana* stocks and from a seronegative patient. No detectable fluorescence signals were obtained for any of them, indicating the specificity of the assays (Table 3).

**Fig 2:**
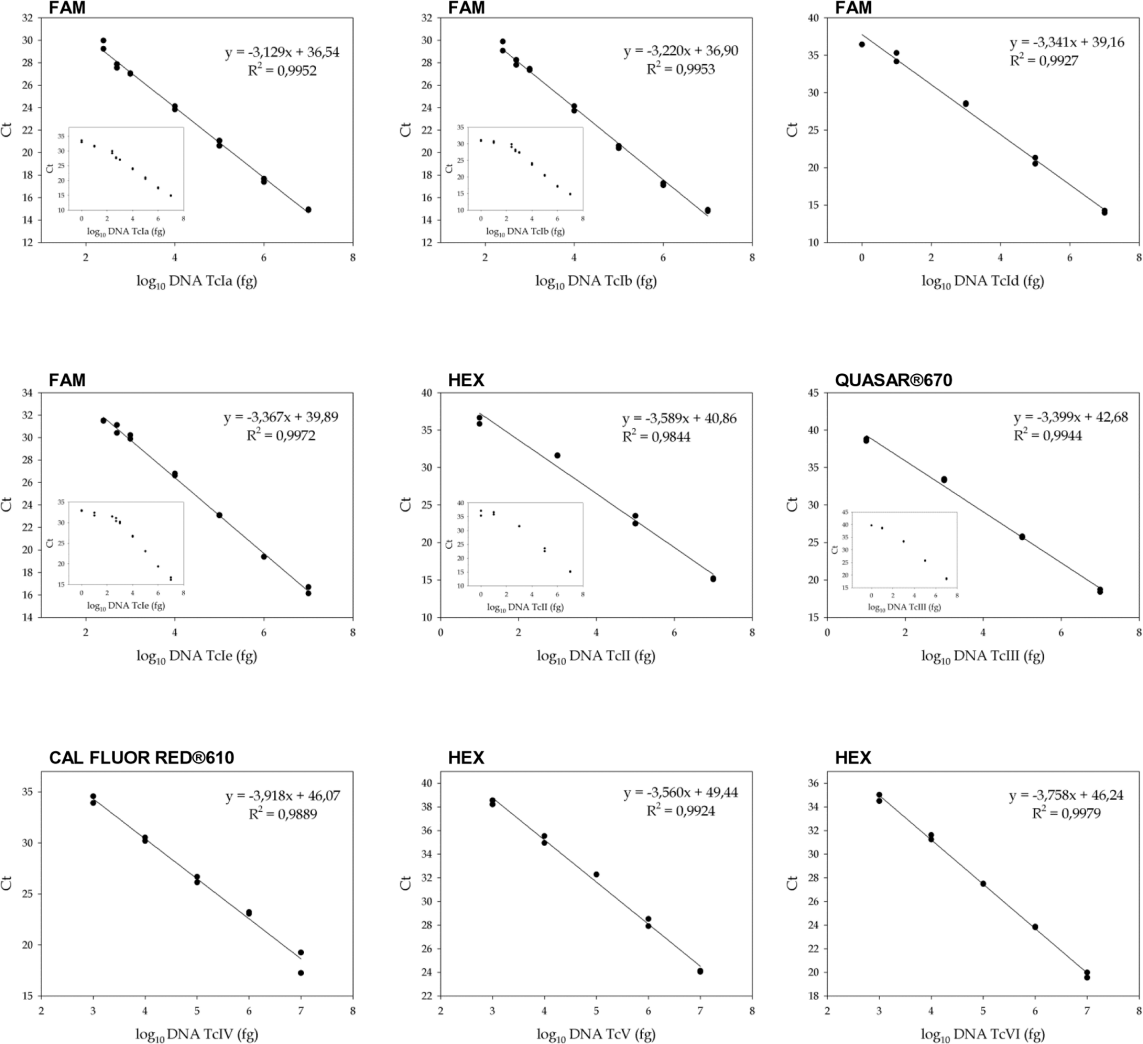
Linear range and analytical sensitivity of the first round SL-IR MTq PCR for *T*. *cruzi* DTUs and TcI SL-IR genotypes. X-axis represents serial dilutions of whole genomic DNA from each stock and Y-axis represents the obtained Ct value. Linear regression analysis, equation and R^2^ are shown for each graph. Inserts inside plots represent the Ct values obtained for the complete DNA concentration range tested (1 fg—10 ng/ reaction tube). TcIa, strain K98; TcIb, strain Cas16; TcId, strain G; TcIe, strain PALV1 cl1; TcII, strain Tu18; TcIII, strain M5631; TcIV, strain CanIII; TcV, strain PAH265; and TcVI, strain CL Brener.

**Fig 3:**
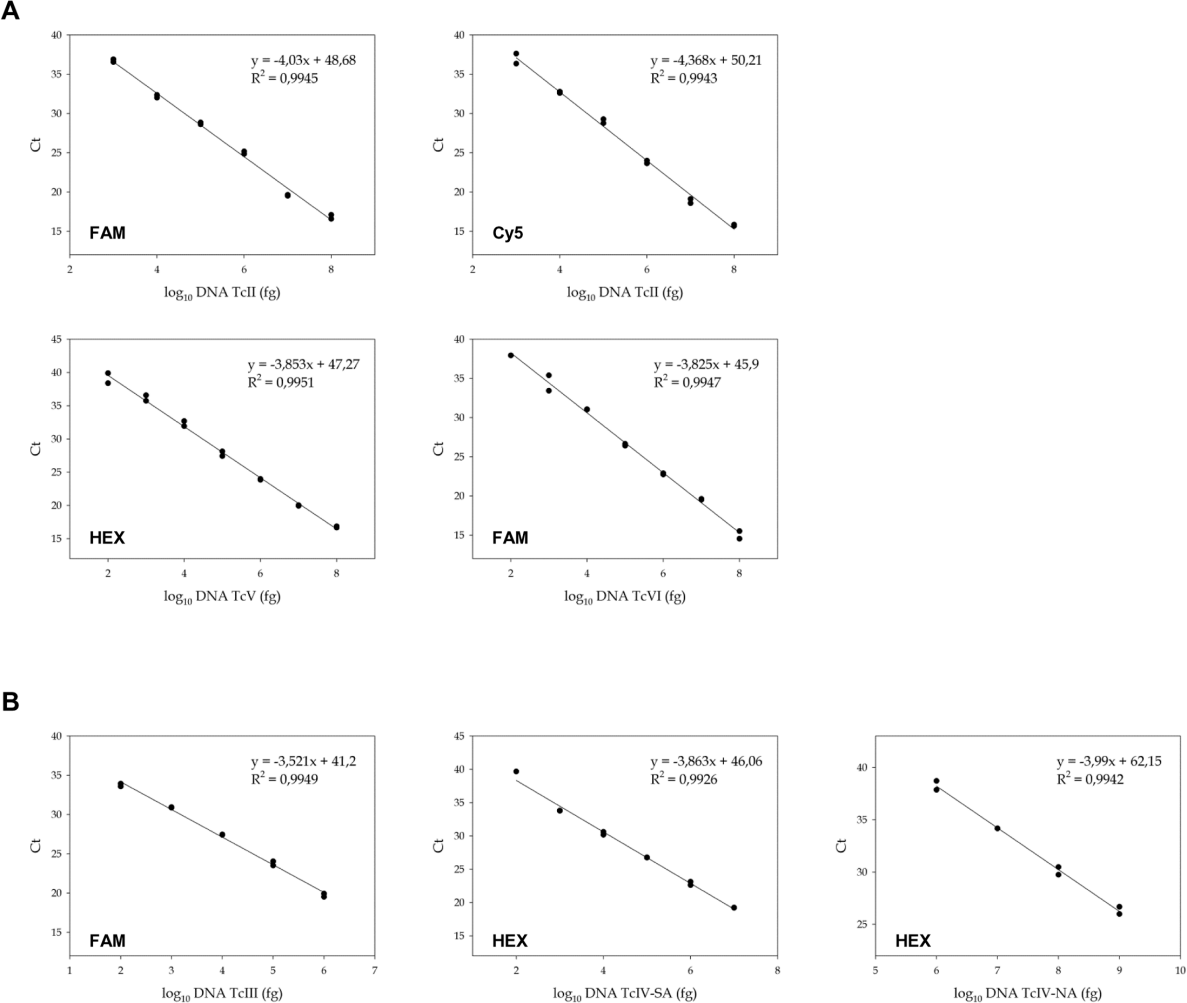
Linear range and analytical sensitivity of the second round multiplex real-time PCR tests. **A.** 18S-COII MTq PCR assay for reference stocks representing *T*. *cruzi* DTUs TcII, TcV and TcVI. Detection of TcII stock is shown for both TaqMan probes 18S-FAM and COII-Cy5. **B.** 24Sα MTq PCR for reference stocks representing *T*. *cruzi* DTUs TcIII, TcIV-SA and TcIV-NA. X-axis represents serial dilutions of whole genomic DNA from each stock and Y-axis represents the obtained Ct value. Linear regression analysis, equation and R^2^ are shown for each graph. TcII, strain Tu18; TcV, strain PAH265; TcVI, strain CL Brener; TcIII, strain M5631; TcIV-SA (TcIV from South America), strain CanIII; TcIV-NA (TcIV from North America), strain Griffin.

Analytical sensitivity and reaction efficiency were estimated separately for each of the three MTq-PCR reactions using genomic DNA from reference stocks representing the six *T*. *cruzi* DTUs: TcIa (K98), TcIb (Cas16), TcId (G), TcIe (PALV1 cl1), TcII (Tu18), TcIII (M5631), TcIV-SA (CanIII), TcIV-NA (Griffin), TcV (PAH265) and TcVI (CL-Brener). The SL-IR MTq-PCR yielded a positive result starting from 1 fg DNA/reaction tube of TcI reference strains with an efficiency (Eff) of 108% (TcIa), 104% (TcIb), 99% (TcId) and 98% (TcIe). Similar sensitivity was obtained for strains representing TcII (Eff: 90%) and TcIII (Eff: 97%). In the cases of TcIV-SA, TcV and TcVI, sensitivity was lower (1 pg DNA/reaction tube) with Eff of 80%, 88% and 86%, respectively (Fig 2). The 18S-COII MTq-PCR reaction rendered a sensitivity of 100 fg DNA/reaction tube for strains representing TcV (Eff: 82%) and TcVI (Eff: 83%) and 1 pg DNA/reaction tube for TcII (Eff: 77% and 70% using the 18S-FAM and the COII-Cy5, respectively) (Fig 3A). The 24Sα-III/IV MTq-PCR method was capable of detecting 100 fg DNA/reaction tube of the TcIII (Eff: 92%) and TcIV-SA (Eff: 81%) stocks, whereas TcIV-NA was detected at concentrations ≥ 1 ng/reaction tube (Eff: 78%) (Fig 3B).

### Evaluation of the Multiplex Real-Time PCR Assays in Biological Samples

A total of 307 biological specimens, including clinical samples (n = 132) as well as samples obtained from different species of vectors (n = 104) and mammal reservoirs (n = 71) from different endemic regions were evaluated using MTq-PCR and a conventional PCR based strategy [11, 17].

#### Clinical samples

Chagas disease patients were classified into five groups according to their infection phase or infection route: AI, ACD, SCD, CI and RCD (see Materials and Methods). From one RCD patient, more than one sample (blood and skin biopsy samples) was available for analysis. The AI group included 10 peripheral blood samples from people who acquired the infection in oral outbreaks in the Amazon region of Bolivia, Venezuela, Colombia and French Guiana; one sample from a vector-transmitted acute patient from Chiapas, Mexico; and two samples from patients who acquired *T*. *cruzi* infection due to organ transplantation. The MTq-PCR was able to characterize DTUs in all AI samples, in total agreement with the conventional techniques, confirming 5 TcI and 6 TcIV cases (Table 4 and S1 Table). In two samples, conventional PCR was not able to discriminate between pure TcV infections or a mixture of TcV plus TcVI. However, the MTq-PCR confirmed TcV, allowing exclusion of TcVI (Table 4 and S1 Table).

**Table 4:** Multiplex real-time PCR genotyping algorithm validation with biological samples.

Samples		Conventional PCR pos	MTq PCR pos	TcI	TcII/V/VI	TcII/VI	TcII	TcIII	TcIV	TcV	TcVI	Mixed infections
**Human**	**AI**	13	13	5	0	0	0	0	6	2	0	0
	**ACD**	64	21	19	0	1	0	0	0	1	0	0
	**SCD**	27	6	6	0	0	0	0	0	0	0	0
	**CI**	16	10	1	1	1	0	0	0	6	0	1^d^
	**RCD** ^a^	12	6	3	0	1	1	0	0	1	0	0
**Vectors**	**Direct sample** ^b^	88	71	47	0	0	1	5	8	1	0	9^e^
	**Culture**	16	15	11	0	0	0	0	4	0	0	0
**Animal reservoirs**	**Direct sample** ^c^	44	41	40	0	0	0	0	0	0	0	1^f^
	**Culture**	27	27	3	0	6	0	16	2	0	0	0
**Total**		307	210	135	1	0	3	21	20	11	8	11

Positive results obtained with the Real-Time and conventional PCR assays for the DTU characterization of biological samples were compared. The number of samples belonging to each DTU group corresponds to the Real-Time PCR algorithm results.

^a^Eleven peripheral blood samples and one skin biopsy sample

^b^Sixty three urine/feces samples on filter paper and 25 abdomen/tissue samples

^c^Forty peripheral blood and 4 heart explant samples; pos, positive results. Mixed infections were characterized as ^d^TcV plus TcVI,

^e^6 TcI plus TcIV, 1 TcI plus TcIII/IV and 2 TcIII plus TcIV,

^f^TcI plus TcII. AI, acute *T*. *cruzi* infection; ACD, asymptomatic chronic Chagas disease; SCD, symptomatic chronic Chagas disease; CI, congenitally infected children; and RCD, patients with reactivation in the context of immunosuppression.

The DTUs present in 10 out of 16 (62.5%) peripheral blood samples analyzed from CI children could be identified by the MTq-PCR. Results were clearly consistent with those obtained by the conventional strategies, confirming 1 TcI and 4 TcV infections (Table 4 and S1 Table). In 4 samples, conventional PCR was not able to discriminate between pure TcV infections or a mixture of TcV plus TcVI. However, the MTq-PCR confirmed TcV in two cases, classified one as an indeterminate TcII/V/VI sample, and classified the remaining one as a mixed infection of TcV plus TcVI. Furthermore, one sample that was classified as indeterminate TcII/V/VI using the conventional PCR algorithm was classified as TcVI using the MTq-PCR (Table 4 and S1 Table).

Eleven peripheral blood samples and one skin biopsy sample from RCD patients were analyzed and six (50%) could be genotyped by MTq-PCR, confirming three as infected with TcI populations (Table 4 and S1 Table). In one sample, conventional PCR was not able to discriminate between pure TcV or a mixture of TcV plus TcVI. However, MTq-PCR confirmed TcV and excluded TcVI. Additionally, the skin biopsy sample was classified as doubtful TcII/VI by the conventional PCR, but MTq-PCR confirmed the presence of TcII DNA. On the other hand, *T*. *cruzi* populations in the peripheral blood sample of the above mentioned patient were confirmed as belonging to TcII by the conventional method and classified as indeterminate TcII/VI by MTq-PCR (Table 4 and S1 Table).

A low proportion of chronic Chagas disease patients’ samples (32.8% ACD and 22.2% SCD) could be characterized by the MTq-PCR, and most of them were typed as TcI (n = 25), in full accordance with conventional typing (Table 4 and S1 Table). In one sample, conventional PCR was not able to discriminate between pure TcV infection or a mixture of TcV plus TcVI. However, TaqMan PCR confirmed TcV and eliminated TcVI. On the other hand, another sample was confirmed as TcVI by the conventional PCR method and classified as indeterminate TcII/VI by the MTq-PCR (Table 4 and S1 Table).

#### Triatomine samples

A total of 104 samples (88 direct samples and 16 culture isolates) obtained from urine, feces and tissue (midgut/abdomen) specimens from triatomines were processed. The MTq-PCR gave positive results in 80.7% and 93.8% of direct samples and isolated cultures, respectively, confirming 54 TcI, 1 TcII, 2 TcIII and 8 TcIV (Tables 4 and S2). Overall typed vector samples, two indeterminate TcIII (or TcIII plus TcI) and one TcV (or TcV plus TcVI) specimens by the conventional methods were confirmed as TcIII, TcI and TcV, respectively, by the MTq-PCR (Tables 4 and S2).

#### Reservoir samples

The study included 71 samples (44 direct samples and 27 culture isolates) obtained from peripheral blood and tissue specimens from mammal reservoirs of *T*. *cruzi*, most of which were successfully typified by MTq-PCR (100% of culture isolates and 93.2% of direct samples), confirming 28 TcI, 16 TcIII and 1 TcIV (Tables 4 and S3). Six TcVI samples obtained from peripheral blood of *Canis familiaris* were classified as indeterminate TcII/VI by MTq-PCR (Tables 4 and S3).

#### Analysis of mixed infections

In clinical samples, a mixed infection by TcI plus TcII/V/VI found in a SCD patient gave no amplification after SL-IR MTq-PCR, probably because of its very low parasitic load [47].

Detection of DTU-mixed infections in vector samples revealed a complex situation, in which MTq-PCR succeeded in resolving six out of 16 mixtures previously characterized by the conventional PCR tests; nine were characterized as single infections and one gave negative results (Tables 4 and S2). On the other hand, three samples that were classified as inconclusive TcIII (or TcIII plus TcI) using conventional PCR were classified as mixed infections using MTq-PCR (2 TcIII plus TcIV and 1 TcI plus TcIII/IV) (Tables 4 and S2).

In the case of reservoir samples, MTq-PCR confirmed one case of TcI plus TcII mixed infection but failed to resolve 17 other mixed infections previously characterized in *Didelphis virginiana*, *Macaca fascicularis*, *Canis familiaris* and *Felis catus* by conventional PCR (Tables 4 and S3).

## Discussion

As a consequence of the standardized nomenclature for the six *T*. *cruzi* DTUs having been ratified by a committee of experts [6], it became imperative to develop a reliable genotyping strategy that could be adopted by the research community [8]. Throughout the past years, several typing schemes have been developed. A PCR assay system based on the amplification of particular regions of the SL gene and 24Sα-rDNA [44] and 18S rDNA [48] was first proposed [15] in which the size polymorphisms of the amplification products were suitable for *T*. *cruzi* assignment into each of the six DTUs. A multilocus PCR-RFLP analysis of genetic polymorphism of 12 loci also was proposed for DTU genotyping [16]. Additionally, a three-marker sequential typing strategy was proposed consisting of PCR amplification of the 24Sα-rDNA and PCR-RFLP of the heat shock protein 60 and glucose-6-phosphate isomerase loci [18]. Yeo et al. (2011) and Lauthier et al. (2012) designed Multilocus Sequence Typing (MLST) schemes in which sequence information of 4 to 10 single copy housekeeping genes allowed the resolution of the six DTUs [21–22]. A recent assay that uses a single copy gene (TcSC5D) followed by two RFLP reactions has been reported [23]. However, most of the above mentioned assays are complex to perform and have been applied only to cultured parasites. Another scheme using nested-hot-start PCR assays allows direct DTU typing in biological [25, 49] and clinical [11, 17] samples but requires between 3 and 9 sequential PCR reactions.

To overcome these difficulties we developed a novel MTq-PCR approach that identifies the six *T*. *cruzi* DTUs in a single or two sequential reactions with adequate sensitivity to analyze different types of biological samples, such as those derived from triatomine vectors and different type of wildlife, livestock, pets and human tissues. The Real-Time format reduces PCR associated contamination and is amenable to quantification, automation and kit production. A first round allows distinction of TcI strains from those belonging to TcIII/IV or TcII/V/VI groups, which are discriminated after a second MTq-PCR round. The method was inclusive for a panel of 39 *T*. *cruzi* stocks. In particular, the TcI primer/probe set was inclusive for all TcI SL-IR genotypes [30], and the TcIV primer/probe set was inclusive for TcIV strains from South and North America [46]. Besides, the test did not recognize human, *T*. *rangeli* and *Leishmania* spp. DNAs.

MTq-PCR methods showed an analytical sensitivity ranging from 1 fg to 1 pg DNA per reaction tube depending on the DTU being analyzed. As an exception, TcIV-NA was detected at concentrations ≥ 1 ng/reaction tube by the 24Sα-III/IV MTq-PCR. The analytical sensitivity for the conventional PCR scheme used in this study was reported in Burgos et al. (2007) [17] and ranged from 100 fg to 10 pg DNA per reaction tube depending on the reaction and the DTU under analysis. Thus, both PCR algorithms used in the present study showed similar ranges of sensitivity when compared at analytical levels.Out of 210 biological samples that could be typed by both algorithms, 24 (11.4%) gave inconclusive TcII/V/VI, TcII/VI, TcV (or TcV plus TcVI) and TcIII (or TcIII plus TcI) results by either conventional or MTq-PCR. In nine samples, conventional PCR was not able to discriminate between single TcV infection and a mixture of TcV plus TcVI. However, MTq-PCR confirmed TcV in seven of these samples thanks to specific detection of the 18S-HEX probe. One sample, typed as TcII/V/VI by conventional PCR, could be resolved as TcII/VI by MTq-PCR. Furthermore, an indeterminate TcII/VI and 2 TcIII (or TcIII plus TcI) samples were confirmed as TcII, TcI and TcIII, respectively, by MTq-PCR (S1–S3 Tables). On the other hand, 7 TcVI and one TcII samples typed by the conventional PCR algorithm were classified as indeterminate TcII/VI by the MTq-PCR (S1–S3 Tables).

Finally, both algorithms confirmed mixed infections in one patient from Jujuy, Argentina (TcV plus TcVI), in one cat from Mexico (TcI plus TcII) and in several sylvatic vector species, such as TcI plus TcIII, TcI plus TcIV and TcIII plus TcIV (S1–S3 Tables). In general the MTq-PCR detected mixed infections in a lesser extent than the conventional PCR scheme. Oligonucleotide interactions, competition for reagents, different amplification efficiency of the targets, and accumulation of amplicons of the predominant target that inhibit Taq polymerase are factors that might be involved.

The MTq-PCR test was less sensitive than conventional PCR algorithm for direct typing of peripheral blood samples of a proportion of chronic Chagas disease patients harboring low parasite loads. We have evaluated the analytical sensitivity of the assay using mixtures of *T*. *cruzi* DNA with DNA extracted from human blood from non-infected subjects and no differences in analytical sensitivity were found (S1 Fig). This suggests that the lower clinical sensitivity of the assay in blood samples would not be due to inhibitory substances present in the samples. In some human cases tested in this study, we can not discard some DNA degradation with respect to the period where the extracts were analyzed using conventional PCR algorithm [50].

The findings herein obtained, promote MTq-PCR as a valuable laboratory tool for distinction of *T*. *cruzi* DTUs. It appears adequate in surveillance and identification of outbreaks sources [51] or to follow-up acute infections of seronegative recipients that receive infected organs from seropositive donors [52].

## Supporting Information

S1 Table DTU characterization of clinical samples using conventional PCR and the multiplex real-time PCR genotyping algorithms. (DOCX)

S2 Table DTU characterization of triatomine samples using conventional PCR and the multiplex real-time PCR genotyping algorithms. (DOCX)

S3 Table DTU characterization of reservoir samples using conventional PCR and the multiplex real-time PCR genotyping algorithms. (DOCX)

S1 Fig Linear range and analytical sensitivity of the first round SL-IR MTq PCR for a TcIa representative stock in both presence and absence of 38 ng DNA extracted from human blood from non-infected subjects. X-axis represents serial dilutions of whole genomic DNA and Y-axis represents the obtained Ct value. TcIa, strain K98.
(TIF)
