# Supplementary material for: Multiplex Real-Time PCR Assay Using TaqMan Probes for the Identification of Trypanosoma cruzi DTUs in Biological and Clinical Samples
Source: PLoS Negl Trop Dis. 2015 May 19;9(5):e0003765. doi: 10.1371/journal.pntd.0003765 (PMC4437652; doi:10.1371/journal.pntd.0003765)
Supplement: S1 Table — (DOCX) [file pntd.0003765.s001.docx]

**Table S1.**

| **ID** | **Clinical stage** | **Geographical origin** | **Sample type** | **Extraction method** | **DTU** | | **Reference** |
| --- | --- | --- | --- | --- | --- | --- | --- |
|  |  |  |  |  | **cPCR** | **MTq-PCR** |  |
| **2384-2007** | AI (oral outbreak) | Chacao, Venezuela | Blood | 1 | TcI | TcI | a |
| **2389-2007** | AI (oral outbreak) | Chacao, Venezuela | Blood | 1 | TcI | TcI | a |
| **VTH** | AI (oral outbreak) | Iracoubo, French Guiana | Blood | 2 | TcId | TcI | b, c |
| **VTV** | AI (oral outbreak) | Iracoubo, French Guiana | Blood | 2 | TcI | TcI | b,c |
| **1** | AI (oral outbreak) | Guayamerín, Bolivia | Blood | 3 | TcIV | TcIV | d |
| **2** | AI (oral outbreak) | Guayamerín, Bolivia | Blood | 3 | TcIV | TcIV | d |
| **7** | AI (oral outbreak) | Guayamerín, Bolivia | Blood | 3 | TcIV | TcIV | d |
| **10** | AI (oral outbreak) | Guayamerín, Bolivia | Blood | 3 | TcIV | TcIV | d |
| **15** | AI (oral outbreak) | Guayamerín, Bolivia | Blood | 3 | TcIV | TcIV | d |
| **16** | AI (oral outbreak) | Guayamerín, Bolivia | Blood | 3 | TcIV | TcIV | d |
| **122012** | AI (vectorial) | Mazatan, Chiapas, Mexico | Blood | 4 | TcI | TcI | d |
| **NA** | AI (Tx) | Santiago del Estero, Argentina (donor) | Blood | 1 | TcV or TcV + TcVI | TcV | e |
| **CJ** | AI (Tx) | Tucumán, Argentina (donor) | Blood | 1 | TcV or TcV + TcVI | TcV | e |
| **106** | CI | Corrientes, Argentina | Blood | 5 | TcV | neg | f |
| **801-HE** | CI | Chaco, Argentina | Blood | 6 | TcV | TcV | d |
| **802-HT** | CI | Chaco, Argentina | Blood | 6 | TcV | TcV | d |
| **26RN** | CI | Jujuy, Argentina | Blood | 3 | TcII/V/VI | TcVI (TcVI/II) | d |
| **RN56** | CI | Jujuy, Argentina | Blood | 3 | TcV | TcV | d |
| **36C** | CI | Jujuy, Argentina | Blood | 3 | TcV or TcV + TcVI | TcV | d |
| **37C** | CI | Jujuy, Argentina | Blood | 3 | TcV or TcV + TcVI | TcV + TcVI (TcVI/II) | d |
| **48RN** | CI | Jujuy, Argentina | Blood | 3 | TcV or TcV + TcVI | TcII/V/VI | d |
| **RN96** | CI | Jujuy, Argentina | Blood | 3 | TcV or TcV + TcVI | TcV | d |
| **5399-GM** | CI | Argentina | Blood | 6 | TcV | neg | d |
| **814C** | CI | Bolivia | Blood | 6 | TcI | TcI | d |
| **5460-FC** | CI | Bolivia | Blood | 6 | TcV | TcV | d |
| **5461-FJ** | CI | Bolivia | Blood | 6 | TcV | neg | d |
| **5470-CBo** | CI | Bolivia | Blood | 6 | TcV | neg | d |
| **5487-CBr** | CI | Bolivia | Blood | 6 | TcV | neg | d |
| **5086-ZF** | CI | no data | Blood | 6 | TcV | neg | d |
| **LB 498** | ACD | Chaco, Argentina | Blood | 5 | TcI | neg | f |
| **Q31** | ACD | Chaco, Argentina | Blood | 5 | TcII/V/VI | neg | f |
| **Q26** | ACD | Chaco, Argentina | Blood | 5 | TcV | neg | f |
| **Q28** | ACD | Chaco, Argentina | Blood | 5 | TcV | neg | f |
| **Q29** | ACD | Chaco, Argentina | Blood | 5 | TcV | neg | f |
| **Q30** | ACD | Chaco, Argentina | Blood | 5 | TcV | neg | f |
| **Q32** | ACD | Chaco, Argentina | Blood | 5 | TcV | neg | f |
| **Q34** | ACD | Chaco, Argentina | Blood | 5 | TcV | neg | f |
| **38** | ACD | Chaco, Argentina | Blood | 5 | TcII/V/VI | neg | f |
| **39** | ACD | Chaco, Argentina | Blood | 5 | TcII/V/VI | neg | f |
| **40** | ACD | Chaco, Argentina | Blood | 5 | TcII/V/VI | neg | f |
| **84** | ACD | Chaco, Argentina | Blood | 5 | TcII/V/VI | neg | f |
| **42** | ACD | Chaco, Argentina | Blood | 5 | TcV | neg | f |
| **64** | ACD | Chaco, Argentina | Blood | 5 | TcV | neg | f |
| **67** | ACD | Chaco, Argentina | Blood | 5 | TcV | neg | f |
| **LH 232** | ACD | Chaco, Argentina | Blood | 5 | TcII/V/VI | neg | f |
| **LH 222** | ACD | Chaco, Argentina | Blood | 5 | TcV | neg | f |
| **LH 215** | ACD | Chaco, Argentina | Blood | 5 | TcV or TcV + TcII/VI | neg | f |
| **VL3** | ACD | Chaco, Argentina | Blood | 5 | TcV | neg | f |
| **VL5** | ACD | Chaco, Argentina | Blood | 5 | TcV | neg | f |
| **M4** | ACD | Chaco, Argentina | Blood | 5 | TcV | neg | f |
| **G53** | ACD | Chaco, Argentina | Blood | 5 | TcV | neg | f |
| **NP 266** | ACD | Chaco, Argentina | Blood | 5 | TcV | neg | f |
| **HE 237** | ACD | Corrientes, Argentina | Blood | 5 | TcV | neg | f |
| **HE 238** | ACD | Corrientes, Argentina | Blood | 5 | TcV | neg | f |
| **HE 198** | ACD | Corrientes, Argentina | Blood | 5 | TcV or TcV + TcVI | neg | f |
| **HE 392** | ACD | Corrientes, Argentina | Blood | 5 | TcV or TcV + TcVI | TcV | f |
| **412E** | ACD | Sucre, Bolivia | Blood | 6 | TcI | TcI | d |
| **1586B** | ACD | Sucre, Bolivia | Blood | 6 | TcI | TcI | d |
| **13263A** | ACD | Sucre, Bolivia | Blood | 6 | TcI | neg | d |
| **2492C** | ACD | Cochabamba, Bolivia | Blood | 6 | TcI | neg | d |
| **2868C** | ACD | Cochabamba, Bolivia | Blood | 6 | TcI | TcI | d |
| **7940** | ACD | Cochabamba, Bolivia | Blood | 6 | TcI | TcI | d |
| **12979(2)** | ACD | Cochabamba, Bolivia | Blood | 6 | TcI | TcI | d |
| **19988(9)** | ACD | Cochabamba, Bolivia | Blood | 6 | TcIa | neg | d |
| **22987(3)** | ACD | Cochabamba, Bolivia | Blood | 6 | TcI | TcI | d |
| **4965B** | ACD | Cochabamba, Bolivia | Blood | 6 | TcVI | TcVI (VI/II) | d |
| **4965A** | ACD | Chuquisaca, Bolivia | Blood | 6 | TcI | neg | d |
| **6711(A)** | ACD | Santa Cruz, Bolivia | Blood | 6 | TcI | neg | d |
| **7752** | ACD | Santa Cruz, Bolivia | Blood | 6 | TcI | TcI | d |
| **19989(6)** | ACD | Santa Cruz, Bolivia | Blood | 6 | TcI | TcI | d |
| **14078(5)** | ACD | Tarija, Bolivia | Blood | 6 | TcIa | TcI | d |
| **15842(8)** | ACD | Tarija, Bolivia | Blood | 6 | TcIa | neg | d |
| **11612A** | ACD | La Paz, Bolivia | Blood | 6 | TcI | TcI | d |
| **140079(4)** | ACD | Potosí, Bolivia | Blood | 6 | TcIa | TcI | d |
| **4194C** | ACD | Bolivia | Blood | 6 | TcI | neg | d |
| **16847E** | ACD | Bolivia | Blood | 6 | TcI | neg | d |
| **23467B** | ACD | Bolivia | Blood | 6 | TcI | TcI | d |
| **24544A** | ACD | Bolivia | Blood | 6 | TcI | TcI | d |
| **3A** | ACD | Bolivia | Blood | 6 | TcI | neg | d |
| **7A** | ACD | Bolivia | Blood | 6 | TcI | TcI | d |
| **9A** | ACD | Bolivia | Blood | 6 | TcI | neg | d |
| **11A** | ACD | Bolivia | Blood | 6 | TcI | neg | d |
| **16A** | ACD | Bolivia | Blood | 6 | TcI | TcI | d |
| **19A** | ACD | Bolivia | Blood | 6 | TcI | TcI | d |
| **25A** | ACD | Bolivia | Blood | 6 | TcI | TcI | d |
| **28C** | ACD | Bolivia | Blood | 6 | TcI | TcI | d |
| **30A** | ACD | Bolivia | Blood | 6 | TcI | TcI | d |
| **108a** | ACD | Bolivia | Blood | 6 | TcI | neg | d |
| **YQC** | ACD | Bolivia | Blood | 6 | TcI | neg | d |
| **EC** | ACD | Bolivia | Blood | 6 | TcI | neg | d |
| **16214F** | ACD | Bolivia | Blood | 6 | TcII/V/VI | neg | d |
| **17530E** | ACD | Bolivia | Blood | 6 | TcII/V/VI | neg | d |
| **20805D** | ACD | Bolivia | Blood | 6 | TcII/V/VI | neg | d |
| **LH 202** | SCD (heart disease) | Chaco, Argentina | Blood | 5 | TcII/V/VI | neg | f |
| **LH 200** | SCD (heart disease) | Chaco, Argentina | Blood | 5 | TcV | neg | f |
| **LH 201** | SCD (heart disease) | Chaco, Argentina | Blood | 5 | TcV | neg | f |
| **LH 211** | SCD (heart disease) | Chaco, Argentina | Blood | 5 | TcV | neg | f |
| **LH 231** | SCD (heart disease) | Chaco, Argentina | Blood | 5 | TcV | neg | f |
| **M13** | SCD (heart disease) | Chaco, Argentina | Blood | 5 | TcV | neg | f |
| **M17** | SCD (heart disease) | Chaco, Argentina | Blood | 5 | TcV | neg | f |
| **M20** | SCD (heart disease) | Chaco, Argentina | Blood | 5 | TcV | neg | f |
| **M358** | SCD (heart disease) | Chaco, Argentina | Blood | 5 | TcV or TcV + TcVI | neg | f |
| **32C** | SCD (heart disease) | Argentina | Blood | 6 | TcI | TcI | d |
| **7991(1)** | SCD (heart disease) | Sucre, Bolivia | Blood | 6 | TcI | neg | d |
| **4A** | SCD (heart disease) | Bolivia | Blood | 6 | TcI | neg | d |
| **13A** | SCD (heart disease) | Bolivia | Blood | 6 | TcI | neg | d |
| **14A** | SCD (heart disease) | Bolivia | Blood | 6 | TcI | TcI | d |
| **31A** | SCD (heart disease) | Bolivia | Blood | 6 | TcI | TcI | d |
| **40b** | SCD (heart disease) | Bolivia | Blood | 6 | TcI | neg | d |
| **42c** | SCD (heart disease) | Bolivia | Blood | 6 | TcI | neg | d |
| **43a** | SCD (heart disease) | Bolivia | Blood | 6 | TcI | neg | d |
| **DN** | SCD (heart disease) | Bolivia | Blood | 6 | TcI + TcII/V/VI | neg | d |
| **4880C** | SCD (digestive disease) | Santa Cruz, Bolivia | Blood | 6 | TcI | TcI | d |
| **11471C** | SCD (digestive disease) | Cochabamba, Bolivia | Blood | 6 | TcIa | TcI | d |
| **222018(1)** | SCD (digestive disease) | Cochabamba, Bolivia | Blood | 6 | TcI | neg | d |
| **44a** | SCD (digestive disease) | Bolivia | Blood | 6 | TcI | neg | d |
| **120a** | SCD (digestive disease) | Bolivia | Blood | 6 | TcI | neg | d |
| **7942** | SCD (digestive and heart disease) | Chuquisaca, Bolivia | Blood | 6 | TcI | TcI | d |
| **13618(2)** | SCD (digestive and heart disease) | Cochabamba, Bolivia | Blood | 6 | TcIa | neg | d |
| **18140** | SCD (digestive and heart disease) | Santa Cruz, Bolivia | Blood | 6 | TcI | neg | d |
| RR | RCD (Tx) | Buenos Aires, Argentina | Blood | 1 | TcV | neg | d |
| VL | RCD (Tx) | Catamarca, Argentina | Blood | 1 | TcV | neg | d |
| LM | RCD (Tx) | Catamarca, Argentina | Blood | 1 | TcIa | TcI | d |
| CHA | RCD (Tx) | Chaco, Argentina | Blood | 1 | TcV | neg | d |
| BJ | RCD (Tx) | La Pampa, Argentina | Blood | 1 | TcII | TcII (TcII/VI) | d |
|  |  |  | Tissue (skin) | 1 | TcII/VI | TcII | d |
| CD | RCD (Tx) | Salta, Argentina | Blood | 1 | TcId | TcI | d |
| AMA | RCD (Tx) | Santa Fe, Argentina | Blood | 1 | TcV or TcV + TcVI | neg | d |
| CHL | RCD (Tx) | Santiago del Estero, Argentina | Blood | 1 | TcIa | TcI | d |
| VLR | RCD (Tx) | Santiago del Estero, Argentina | Blood | 1 | TcV or TcV + TcVI | TcV | d |
| FJ | RCD (Tx) | no data | Blood | 1 | TcV | neg | d |
| CL | RCD (Linfoma) | no data | Blood | 1 | TcV or TcV + TcII/VI | neg | d |

1, High Pure PCR Template Preparation Kit (Roche); 2, DNeasy Tissue Kit (QIAGEN); 3, QIAamp DNA mini kit (QIAGEN); 4, DNAzol; 5, CTAB; 6, Phenol-Chloroform; ^a^Muñoz-Calderón et al. (2013); ^b^Cura et al. (2010); ^c^Blanchet et al., submitted; ^d^This work; ^e^Cura et al. (2013), ^f^Cura et al. (2012); DTU, Discrete Typing Unit; neg, negative; cPCR, conventional PCR algorithm; MTq-PCR, multiplex TaqMan Real-Time PCR method; AI, acute *T. cruzi* infected patients; ACD, asymptomatic Chagas disease patients; SCD, symptomatic chronic Chagas disease patients; CI, congenitally infected children; RCD, patients with clinical reactivation in the context of immunosuppression, Tx, transplantation.
