# Supplementary material for: Multiplex Real-Time PCR Assay Using TaqMan Probes for the Identification of Trypanosoma cruzi DTUs in Biological and Clinical Samples
Source: PLoS Negl Trop Dis. 2015 May 19;9(5):e0003765. doi: 10.1371/journal.pntd.0003765 (PMC4437652; doi:10.1371/journal.pntd.0003765)
Supplement: S2 Table — (DOCX) [file pntd.0003765.s002.docx]

**Table S2**

| **ID** | **Geographical origin** | **Vector species** | **Sample type** | **Extraction method** | **DTU** | | **Reference** |
| --- | --- | --- | --- | --- | --- | --- | --- |
|  |  |  |  |  | **cPCR** | **MTq-PCR** |  |
| **197,1** | Monte Alegre do Piauí, Brazil | *Rhodnius neglectus* | Feces | 1 | TcId | neg | a |
| **197,8** | Monte Alegre do Piauí, Brazil | *Rhodnius neglectus* | Feces | 1 | TcId | TcI | a |
| **198,7** | Monte Alegre do Piauí, Brazil | *Rhodnius neglectus* | Feces | 1 | TcId | TcI | a |
| **198,2** | Monte Alegre do Piauí, Brazil | *Rhodnius neglectus* | Tissue (Abdomen) | 2 | TcId | TcI | a |
| **198,3** | Monte Alegre do Piauí, Brazil | *Rhodnius neglectus* | Tissue (Abdomen) | 2 | TcI | neg | a |
| **198,4** | Monte Alegre do Piauí, Brazil | *Rhodnius neglectus* | Tissue (Abdomen) | 2 | TcId + *T. rangeli^a^* | TcI | a |
| **198,7** | Monte Alegre do Piauí, Brazil | *Rhodnius neglectus* | Tissue (Abdomen) | 2 | TcId + *T. rangeli*^a^ | TcI | a |
| **198,8** | Monte Alegre do Piauí, Brazil | *Rhodnius neglectus* | Tissue (Abdomen) | 2 | TcId | TcI | a |
| **290,3** | Posse, Brazil | *Rhodnius neglectus* | Tissue (Abdomen) | 2 | TcI + *T. rangeli*^a^ | neg | a |
| **371** | Taguatinga, Brazil | *Rhodnius neglectus* | Tissue (Abdomen) | 2 | TcI + *T. rangeli^a^* | TcI | a |
| **372** | Taguatinga, Brazil | *Rhodnius neglectus* | Tissue (Abdomen) | 2 | TcId + *T. rangeli^a^* | TcI | a |
| **443** | Palmas, Brazil | *Rhodnius neglectus* | Tissue (Abdomen) | 2 | TcI + *T. rangeli^a^* | neg | a |
| **723,1** | Buritizal, Brazil | *Rhodnius neglectus* | Tissue (Abdomen) | 2 | TcI + *T. rangeli^a^* | neg | a |
| **790** | Ituiutaba, Brazil | *Rhodnius neglectus* | Tissue (Abdomen) | 2 | TcI + *T. rangeli^a^* | neg | a |
| **1154** | Ribeirao Cascalheira, Brazil | *Rhodnius neglectus* | Tissue (Abdomen) | 2 | TcId + *T. rangeli^a^* | TcI | a |
| **RN1153** | Ribeirao Cascalheira, Brazil | *Rhodnius neglectus* | Culture stock | 1 | TcId | neg | a |
| **RN1614** | Quirinopolis, Brazil | *Rhodnius neglectus* | Feces | 1 | TcI | neg | a |
| **J9** | Rémire-Montjoly, French Guiana | *Rhodnius robustus* | Tissue (Abdomen) | 3 | TcI | TcI | b, c |
| **J15** | Rémire-Montjoly, French Guiana | *Rhodnius robustus* | Tissue (Abdomen) | 3 | TcI | TcI | b, c |
| **U10** | Rémire-Montjoly, French Guiana | *Rhodnius robustus* | Feces | 3 | TcI | TcI | b, c |
| **U24** | Rémire-Montjoly, French Guiana | *Rhodnius robustus* | Feces | 3 | TcI | TcI | b, c |
| **U8** | Rémire-Montjoly, French Guiana | *Panstrongylus geniculatus* | Feces | 3 | TcI + TcIII | TcI | b, c |
| **J11** | Rémire-Montjoly, French Guiana | *Panstrongylus geniculatus* | Tissue (Abdomen) | 3 | TcI | TcI | b, c |
| **J36** | Rémire-Montjoly, French Guiana | *Panstrongylus geniculatus* | Tissue (Abdomen) | 3 | TcId | TcI | b, c |
| **J27** | Rémire-Montjoly, French Guiana | *Rhodnius pictipes* | Tissue (Abdomen) | 3 | TcI | TcI | b, c |
| **VV2** | Rémire-Montjoly, French Guiana | *Rhodnius pictipes* | Feces | 3 | TcId | TcI | b, c |
| **J17** | Saint Laurent du Maroni, French Guiana | *Rhodnius robustus* | Tissue (Abdomen) | 3 | TcI | TcI | b, c |
| **J35** | Matoury, French Guiana | *Panstrongylus geniculatus* | Tissue (Abdomen) | 3 | TcI | TcI | b, c |
| **451** | Matoury, French Guiana | *Panstrongylus geniculatus* | Feces | 3 | TcI + TcIII | TcIII | b, c |
| **U43** | Matoury, French Guiana | *Rhodnius pictipes* | Feces | 3 | TcI | TcI | b, c |
| **VV22** | Bélizon, French Guiana | *Panstrongylus lignarius* | Feces | 3 | TcI + TcIV | TcI + TcIV | b, c |
| **J39** | Bélizon, French Guiana | *Rhodnius pictipes* | Tissue (Abdomen) | 3 | TcI | TcI | b, c |
| **VV1** | Cayenne, French Guiana | *Rhodnius robustus* | Feces | 3 | TcId | TcI | b, c |
| **U1** | Cayenne, French Guiana | *Rhodnius robustus* | Feces | 3 | TcI | TcI | b, c |
| **U14** | Cayenne, French Guiana | *Rhodnius robustus* | Feces | 3 | TcI | TcI | b, c |
| **U25** | Cayenne, French Guiana | *Rhodnius robustus* | Feces | 3 | TcI | TcI | b, c |
| **U21** | Cayenne, French Guiana | *Panstrongylus geniculatus* | Feces | 3 | TcI | TcI | b, c |
| **U23** | Cayenne, French Guiana | *Panstrongylus geniculatus* | Feces | 3 | TcI | TcI | b, c |
| **U30** | Cayenne, French Guiana | *Panstrongylus geniculatus* | Feces | 3 | TcIII or TcIII + TcI | TcIII | b, c |
| **U13** | Cayenne, French Guiana | *Rhodnius pictipes* | Feces | 3 | TcI | TcI | b, c |
| **488** | Saül, French Guiana | *Eratyrus mucronatus* | Feces | 3 | TcII | TcII | b, c |
| **495** | Saül, French Guiana | *Panstrongylus geniculatus* | Feces | 3 | TcIII | TcIII | b, c |
| **VV17** | Saül, French Guiana | *Panstrongylus lignarius* | Feces | 3 | TcI + TcIV | TcIV | b, c |
| **480** | Kaw, French Guiana | *Panstrongylus geniculatus* | Feces | 3 | TcIII | TcIII | b, c |
| **481** | Kaw, French Guiana | *Panstrongylus geniculatus* | Feces | 3 | TcIV | TcIV | b, c |
| **441** | Kaw, French Guiana | *Panstrongylus lignarius* | Feces | 3 | TcI + TcIV | TcI + TcIV | b, c |
| **J53** | Iracoubo-Bellevue, French Guiana | *Panstrongylus geniculatus* | Tissue (Abdomen) | 3 | TcIV | undetNEG | b, c |
| **J37** | Roura-Saint Georges, French Guiana | *Rhodnius pictipes* | Tissue (Abdomen) | 3 | TcI + TcIII/IV | TcI | b, c |
| **J45** | Antecum Pata, French Guiana | *Panstrongylus geniculatus* | Tissue (Abdomen) | 3 | TcI + TcIV | TcIV | b, c |
| **512** | Antecum Pata, French Guiana | *Panstrongylus geniculatus* | Feces | 3 | TcIII or TcIII + TcI | TcIII + TcIV | b, c |
| **J47** | Antecum Pata, French Guiana | *Panstrongylus geniculatus* | Tissue (Abdomen) | 3 | TcII + TcI | neg | b, c |
| **U5** | Camopi, French Guiana | *Rhodnius robustus* | Feces | 3 | TcI + TcIV | TcI | b, c |
| **U16** | Macouria, French Guiana | *Panstrongylus geniculatus* | Feces | 3 | TcI + TcIII | TcIII | b, c |
| **452** | Macouria, La Carapa, French Guiana | *Panstrongylus geniculatus* | Feces | 3 | TcIII or TcIII/IV + TcI | TcIII + TcIV | b, c |
| **J55** | French Guiana | *Panstrongylus geniculatus* | Tissue (Abdomen) | 3 | TcIV | neg | b, c |
| **VinG2** | Chaco, Argentina | *Triatoma infestans* | Feces | 4 | TcV | neg | d |
| **V1** | Chaco, Argentina | *Triatoma infestans* | Feces | 4 | TcV | neg | d |
| **Vin G3** | Chaco, Argentina | *Triatoma infestans* | Feces | 4 | TcV or TcV + TcVI | TcV | d |
| **Vin FT** | Chaco, Argentina | *Triatoma infestans* | Feces | 4 | TcV or TcV + TcVI | neg | d |
| **V2** | Chaco, Argentina | *Triatoma infestans* | Feces | 4 | TcII/V/VI | neg | d |
| **V3** | Chaco, Argentina | *Triatoma infestans* | Feces | 4 | TcII/V/VI | neg | d |
| **Vin1** | Chaco, Argentina | *Triatoma infestans* | Feces | 4 | TcII/V/VI | neg | d |
| **AQP 300** | Arequipa, Peru | *Triatoma infestans* | Culture stock | 5 | TcI | TcI | d |
| **TC-01** | Arequipa, Peru | *Triatoma infestans* | Culture stock | 5 | TcI | TcI | d |
| **TC-02** | Arequipa, Peru | *Triatoma infestans* | Culture stock | 5 | TcI | TcI | d |
| **TC-03** | Arequipa, Peru | *Triatoma infestans* | Culture stock | 5 | TcI | TcI | d |
| **TC-04** | Arequipa, Peru | *Triatoma infestans* | Culture stock | 5 | TcI | TcI | d |
| **TC-10** | Arequipa, Peru | *Triatoma infestans* | Culture stock | 5 | TcI | TcI | d |
| **TC-14** | Arequipa, Peru | *Triatoma infestans* | Culture stock | 5 | TcI | TcI | d |
| **TC-15** | Arequipa, Peru | *Triatoma infestans* | Culture stock | 5 | TcI | TcI | d |
| **TC-16** | Arequipa, Peru | *Triatoma infestans* | Culture stock | 5 | TcI | TcI | d |
| **TC59** | Cajamarca, Peru | *Panstrongylus herreri* | Culture stock | 5 | TcId | TcI | d |
| **TC60** | Cajamarca, Peru | *Panstrongylus herreri* | Culture stock | 5 | TcId | TcI | d |
| **TC52** | Cajamarca, Peru | *Panstrongylus herreri* | Culture stock | 5 | TcIV | TcIV | d |
| **Tc54** | Cajamarca, Peru | *Panstrongylus herreri* | Culture stock | 5 | TcIV | TcIV | d |
| **Tc56** | Cajamarca, Peru | *Panstrongylus herreri* | Culture stock | 5 | TcIV | TcIV | d |
| **TC-62** | Cajamarca, Peru | *Panstrongylus herreri* | Culture stock | 5 | TcIV | TcIV | d |
| **C0004** | Palenque, Chiapas, Mexico | *Triatoma dimidiata* | Tissue (Midgut) | 6 | TcIa | TcI | d |
| **C0006** | Palenque, Chiapas, Mexico | *Triatoma dimidiata* | Tissue (Midgut) | 6 | TcI | TcI | d |
| **C0015** | Palenque, Chiapas, Mexico | *Triatoma dimidiata* | Tissue (Midgut) | 6 | TcI | TcI | d |
| **C0016** | Palenque, Chiapas, Mexico | *Panstrongylus rufotuberculatus* | Tissue (Midgut) | 6 | TcIa | TcI | d |
| **C0019** | Palenque, Chiapas, Mexico | *Panstrongylus rufotuberculatus* | Tissue (Midgut) | 6 | TcI | TcI | d |
| **C1080001A** | Salina Cruz, Oaxaca, Mexico | *Triatoma phyllosoma* | Tissue (Midgut) | 6 | TcI | neg | d |
| **C1080018A** | Salina Cruz, Oaxaca, Mexico | *Triatoma phyllosoma* | Tissue (Midgut) | 6 | TcIa | TcI | d |
| **C1080032A** | Salina Cruz, Oaxaca, Mexico | *Triatoma phyllosoma* | Tissue (Midgut) | 6 | TcI | TcI | d |
| **C3Y11** | Xul, Yucatan, Mexico | *Triatoma dimidiata* | Tissue (Midgut) | 6 | TcI | TcI | d |
| **C3Y13** | Cuncunul, Yucatan, Mexico | *Triatoma dimidiata* | Tissue (Midgut) | 6 | TcI | TcI | d |
| **C3Y14** | Cuncunul, Yucatan, Mexico | *Triatoma dimidiata* | Tissue (Midgut) | 6 | TcI | TcI | d |
| **C3J21** | Chamela, Jalisco, Mexico | *Triatoma longipennis* | Tissue (Midgut) | 6 | TcIa | TcI | d |
| **C3NL23** | Bustamante, Nuevo Leon, Mexico | *Triatoma gerstaeckeri* | Tissue (Midgut) | 6 | TcIII or TcIII + TcI | TcI | d |
| **BG08041** | Texas, USA | *Triatoma gerstaeckeri* | Feces | 7 | TcI | TcI | d |
| **Tx324** | Texas, USA | *Triatoma gerstaeckeri* | Feces | 7 | TcIV | TcIV | d |
| **Tx038** | Texas, USA | *Triatoma gerstaeckeri* | Feces | 7 | TcI + TcIII/IV | TcI + TcIV | d |
| **BG10012** | Texas, USA | *Triatoma gerstaeckeri* | Feces | 7 | TcI + TcIII/IV | TcI + TcIV | d |
| **BG08048** | Texas, USA | *Triatoma sanguisuga* | Feces | 7 | TcIa | TcI | d |
| **BG10044** | Texas, USA | *Triatoma lecticularia* | Feces | 7 | TcIV | TcIV | d |
| **Tx011** | Texas, USA | *Triatoma* sp. | Feces | 7 | TcIII or TcIII/IV + TcI | TcI + TcIII/IV | d |
| **BG10043** | California, USA | *Triatoma protracta* | Feces | 7 | TcIa | TcI | d |
| **TX067** | USA | *Triatoma* sp. | Feces | 7 | TcI | TcI | d |
| **TX017** | USA | *Triatoma* sp. | Feces | 7 | TcIV | TcIV | d |
| **TX08019** | USA | *Triatoma* sp. | Feces | 7 | TcI + TcIII/IV | TcI + TcIV | d |
| **TX023** | USA | *Triatoma* sp. | Feces | 7 | TcI + TcIII/IV | TcI + TcIV | d |
| **TXBG08015** | USA | *Triatoma* sp. | Feces | 7 | TcI + TcIV | TcIV | d |
| **BG0814** | USA | *Triatoma* sp. | Feces | 7 | TcI + TcIV | TcIV | d |

1, Machado et al. (2000); 2, High Pure PCR Template Preparation Kit (Roche); 3, DNeasy blood and Tissue kit (QIAGEN); 4, CTAB; 5, Phenol-Chloroform; 6, DNAzol; 7, PURE-GENE DNA Purification Kit (Gentra); ^a^Gurgel-Gonçalves et al., 2012 (*T. rangeli* was identified using a separate PCR reaction); ^b^Péneau et al. (2014); ^c^Péneau, PhD Thesis Université des Antilles et de la Guyane (2014); ^d^This work; DTU, Discrete Typing Unit; neg, negative; cPCR, conventional PCR algorithm; MTq-PCR, multiplex TaqMan Real-Time PCR method.
