# Supplementary material for: Multiplex Real-Time PCR Assay Using TaqMan Probes for the Identification of Trypanosoma cruzi DTUs in Biological and Clinical Samples
Source: PLoS Negl Trop Dis. 2015 May 19;9(5):e0003765. doi: 10.1371/journal.pntd.0003765 (PMC4437652; doi:10.1371/journal.pntd.0003765)
Supplement: S3 Table — (DOCX) [file pntd.0003765.s003.docx]

**Table S3**

| **ID** | **Geographical origin** | **Reservoir species** | **Sample type** | **Extraction method** | **DTU** | | **Reference** |
| --- | --- | --- | --- | --- | --- | --- | --- |
|  |  |  |  |  | **cPCR** | **MTq-PCR** |  |
| **Dn1** | Gran Chaco region, Argentina | *Dasypus novemcinctus* | Culture stock | 1 | TcIII | TcIII | a |
| **Dn27** | Gran Chaco region, Argentina | *Dasypus novemcinctus* | Culture stock | 1 | TcIII | TcIII | a |
| **Dn33** | Gran Chaco region, Argentina | *Dasypus novemcinctus* | Culture stock | 1 | TcIII | TcIII | a |
| **Dn43** | Gran Chaco region, Argentina | *Dasypus novemcinctus* | Culture stock | 1 | TcIII | TcIII | a |
| **Dn44** | Gran Chaco region, Argentina | *Dasypus novemcinctus* | Culture stock | 1 | TcIII | TcIII | a |
| **Dn129** | Gran Chaco region, Argentina | *Dasypus novemcinctus* | Culture stock | 1 | TcIII | TcIII | a |
| **Dn246** | Gran Chaco region, Argentina | *Dasypus novemcinctus* | Culture stock | 1 | TcIII | TcIII | a |
| **Dn274** | Gran Chaco region, Argentina | *Dasypus novemcinctus* | Culture stock | 1 | TcIII | TcIII | a |
| **Dn322** | Gran Chaco region, Argentina | *Dasypus novemcinctus* | Culture stock | 1 | TcIII | TcIII | a |
| **Dn374** | Gran Chaco region, Argentina | *Dasypus novemcinctus* | Culture stock | 1 | TcIII | TcIII | a |
| **Dn438** | Gran Chaco region, Argentina | *Dasypus novemcinctus* | Culture stock | 1 | TcIII | TcIII | a |
| **Dn446** | Gran Chaco region, Argentina | *Dasypus novemcinctus* | Culture stock | 1 | TcIII | TcIII | a |
| **Dn464** | Gran Chaco region, Argentina | *Dasypus novemcinctus* | Culture stock | 1 | TcIII | TcIII | a |
| **Perro 81** | Gran Chaco region, Argentina | *Canis familiaris* | Culture stock | 1 | TcIII | TcIII | b |
| **Perro 266** | Gran Chaco region, Argentina | *Canis familiaris* | Culture stock | 1 | TcIII | TcIII | b |
| **Capitan** | Gran Chaco region, Argentina | *Canis familiaris* | Culture stock | 1 | TcVI | TcVI (TcVI/II) | c |
| **Cual II** | Gran Chaco region, Argentina | *Canis familiaris* | Culture stock | 1 | TcVI | TcVI (TcVI/II) | c |
| **Lamber** | Gran Chaco region, Argentina | *Canis familiaris* | Culture stock | 1 | TcVI | TcVI (TcVI/II) | c |
| **Nico** | Gran Chaco region, Argentina | *Canis familiaris* | Culture stock | 1 | TcVI | TcVI (TcVI/II) | c |
| **Moña** | Gran Chaco region, Argentina | *Canis familiaris* | Culture stock | 1 | TcVI | TcVI (TcVI/II) | c |
| **Bati** | Gran Chaco region, Argentina | *Canis familiaris* | Culture stock | 1 | TcVI | TcVI (TcVI/II) | c |
| **U01-02** | Rémire Montjoly, French Guiana | *Canis familiaris* | Culture stock | 2 | TcIII | TcIII | d |
| **TC-61** | Arequipa, Peru | *Cavia porcellus* | Culture stock | 3 | TcI | TcI | e |
| **20201** | Texas, USA | *Macaca fascicularis* | Culture stock | 3 | TcI | TcI | e |
| **20444** | Texas, USA | *Macaca fascicularis* | Culture stock | 3 | TcI | TcI | e |
| **20281** | Texas, USA | *Macaca fascicularis* | Tissue (heart) | 3 | TcIa | TcI | e |
| **20567** | Texas, USA | *Macaca fascicularis* | Tissue (heart) | 3 | TcIa | TcI | e |
| **20575** | Texas, USA | *Macaca fascicularis* | Tissue (heart) | 3 | TcIa | TcI | e |
| **20857** | Texas, USA | *Macaca fascicularis* | Tissue (heart) | 3 | TcIa | TcI | e |
| **27375** | Texas, USA | *Macaca fascicularis* | Culture stock | 3 | TcIV | TcIV | e |
| **20177** | Texas, USA | *Macaca fascicularis* | Culture stock | 3 | TcI + TcIV | TcIV | e |
| **36P** | Mérida, Yucatán, Mexico | *Canis familiaris* | Blood | 4 | TcI | TcI | e |
| **41P1** | Mérida, Yucatán, Mexico | *Canis familiaris* | Blood | 4 | TcI | TcI | e |
| **44P2** | Mérida, Yucatán, Mexico | *Canis familiaris* | Blood | 4 | TcI | TcI | e |
| **42P** | Mérida, Yucatán, Mexico | *Canis familiaris* | Blood | 4 | TcIa + TcV/VI | neg | e |
| **G19** | Mérida, Yucatán, Mexico | *Felis catus* | Blood | 4 | TcI | TcI | e |
| **G20** | Mérida, Yucatán, Mexico | *Felis catus* | Blood | 4 | TcI | TcI | e |
| **G23** | Mérida, Yucatán, Mexico | *Felis catus* | Blood | 4 | TcI | neg | e |
| **G28** | Mérida, Yucatán, Mexico | *Felis catus* | Blood | 4 | TcI | TcI | e |
| **G30** | Mérida, Yucatán, Mexico | *Felis catus* | Blood | 4 | TcI | TcI | e |
| **G31** | Mérida, Yucatán, Mexico | *Felis catus* | Blood | 4 | TcI | TcI | e |
| **G43** | Mérida, Yucatán, Mexico | *Felis catus* | Blood | 4 | TcIa | TcI | e |
| **G45A** | Mérida, Yucatán, Mexico | *Felis catus* | Blood | 4 | TcI | TcI | e |
| **G47** | Mérida, Yucatán, Mexico | *Felis catus* | Blood | 4 | TcI | TcI | e |
| **G48** | Mérida, Yucatán, Mexico | *Felis catus* | Blood | 4 | TcI | TcI | e |
| **G49** | Mérida, Yucatán, Mexico | *Felis catus* | Blood | 4 | TcI | TcI | e |
| **G50** | Mérida, Yucatán, Mexico | *Felis catus* | Blood | 4 | TcI | TcI | e |
| **G51** | Mérida, Yucatán, Mexico | *Felis catus* | Blood | 4 | TcI | TcI | e |
| **G52A** | Mérida, Yucatán, Mexico | *Felis catus* | Blood | 4 | TcI | TcI | e |
| **G53** | Mérida, Yucatán, Mexico | *Felis catus* | Blood | 4 | TcI | TcI | e |
| **G124** | Mérida, Yucatán, Mexico | *Felis catus* | Blood | 4 | TcI | TcI | e |
| **G129** | Mérida, Yucatán, Mexico | *Felis catus* | Blood | 4 | TcI | TcI | e |
| **G124B** | Mérida, Yucatán, Mexico | *Felis catus* | Blood | 4 | TcI + TcII/V/VI | TcI | e |
| **G125** | Mérida, Yucatán, Mexico | *Felis catus* | Blood | 4 | TcIa + TcII/V/VI | TcI | e |
| **G127** | Mérida, Yucatán, Mexico | *Felis catus* | Blood | 4 | TcI + TcII/V/VI | TcI | e |
| **G104** | Mérida, Yucatán, Mexico | *Felis catus* | Blood | 4 | TcI + TcII/VI | TcI + TcII | e |
| **G128b** | Mérida, Yucatán, Mexico | *Felis catus* | Blood | 4 | TcI + TcII | TcI | e |
| **Dv2** | Mérida, Yucatán, Mexico | *Didelphis virginiana* | Blood | 4 | TcI + TcII | TcI | e |
| **Dv3** | Molas Yucatán, Mexico | *Didelphis virginiana* | Blood | 4 | TcI | TcI | e |
| **Dv11** | Molas Yucatán, Mexico | *Didelphis virginiana* | Blood | 4 | TcI + TcII/V/VI | TcI | e |
| **Dv12** | Molas Yucatán, Mexico | *Didelphis virginiana* | Blood | 4 | TcI + TcII/V/VI | TcI | e |
| **Dv24** | Molas Yucatán, Mexico | *Didelphis virginiana* | Blood | 4 | TcI + TcII/V/VI | TcI | e |
| **Dv36** | Molas Yucatán, Mexico | *Didelphis virginiana* | Blood | 4 | TcI + TcII/V/VI | TcI | e |
| **Dv5** | Molas Yucatán, Mexico | *Didelphis virginiana* | Blood | 4 | TcI + TcV/VI | TcI | e |
| **DV6** | Molas Yucatán, Mexico | *Didelphis virginiana* | Blood | 4 | TcI + TcV/VI | TcI | e |
| **Dv7** | Molas Yucatán, Mexico | *Didelphis virginiana* | Blood | 4 | TcI + TcV/VI | TcI | e |
| **DV30** | Molas Yucatán, Mexico | *Didelphis virginiana* | Blood | 4 | TcI + TcV/VI | TcI | e |
| **DV31** | Molas Yucatán, Mexico | *Didelphis virginiana* | Blood | 4 | TcIa + TcV/VI | TcI | e |
| **Dv32** | Molas Yucatán, Mexico | *Didelphis virginiana* | Blood | 4 | TcIa + TcV/VI | TcI | e |
| **M0039** | Palenque, Chiapas, Mexico | *Didelphis virginiana* | Tissue (heart) | 5 | TcI | TcI | e |
| **Pe37A** | Chalcatzingo, Morelos, Mexico | *Paramelomys levipes* | Tissue (heart) | 5 | TcI | neg | e |

1, Marcet et al. (2006); 2, DNeasy blood and Tissue kit (QIAGEN); 3, Phenol-Chloroform; 4, DNeasy blood and Tissue kit (QIAGEN) 1 mL of peripheral blood sample; 5, DNAzol; ^a^Alvado-Otegui et al. (2012); ^b^Enriquez et al. (2013); ^c^Cardinal et al. (2008); ^d^Péneau et al. (2014); ^e^This work; DTU, Discrete Typing Unit; neg, negative; cPCR, conventional PCR algorithm; MTq-PCR, multiplex TaqMan Real-Time PCR method.
