# Supplementary figures and images for: Multiplex Real-Time PCR Assay Using TaqMan Probes for the Identification of Trypanosoma cruzi DTUs in Biological and Clinical Samples
Source: PLoS Negl Trop Dis. 2015 May 19;9(5):e0003765. doi: 10.1371/journal.pntd.0003765 (PMC4437652; doi:10.1371/journal.pntd.0003765)

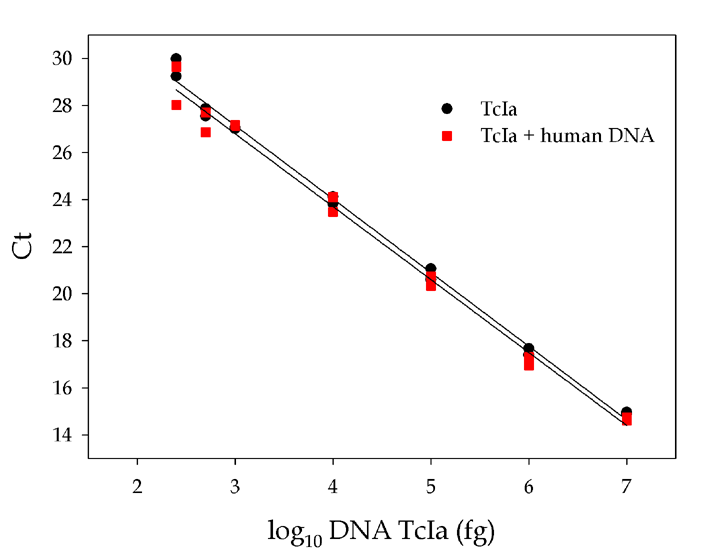

Supplement: S1 Fig — X-axis represents serial dilutions of whole genomic DNA and Y-axis represents the obtained Ct value. TcIa, strain K98. (TIF) [file pntd.0003765.s004.tif]
